# Frequencies of mobilized colistin resistance *(mcr-1, 2*) genes in clinically isolated *Escherichia coli*; a cross sectional study

**DOI:** 10.1186/s13104-023-06455-3

**Published:** 2023-08-31

**Authors:** Bahman Mirzaei, Aida Ebrahimi, Bahareh Hariri, Zahra Sokouti, Niloufar Kazemi, Narges Moradi

**Affiliations:** 1 Department of Medical Microbiology and Virology, School of Medicine, Zanjan University of Medical Sciences, Zanjan, Iran; 2 Student Research Committee, Zanjan University of Medical Sciences, Zanjan, Iran; 3 Department of Life Technologies, University of Turku, Turku, Finland

**Keywords:** *Eshershia coli*, Antibiotic resistant, Colistin

## Abstract

**Objective:**

*Escherichia coli (E. coli)* is an opportunistic bacterium, which is globally recognized for its high prevalence and antimicrobial resistance (AMR). The presence of colistin-resistant representative *mcr*- 1, 2 genes in multi-drug resistant (MDR) clinically isolated *E. coli* is the main goal of this survey. After biochemically and molecular confirmation tests, susceptibility testing, biofilm formation, and minimum inhibitory concentration to colistin were performed on 100 *E. coli* isolates. Subsequently, taking advantage of uniplex-PCR, the presence of some responsible genes (*mcr*- 1, *mcr*- 2) to colistin-resistant phenotypes in mentioned bacterium was assessed.

**Results:**

Disc diffusion methods indicated that the highest resistance rate was against ampicillin (80.0%), and trimethoprim/sulfamethoxazole (63%). Among the *E. coli* isolates, 72 (72.0%) was determined as MDR, respectively. Moreover, 47 (47%) strains were determined as extreme beta-lactamase (ESBL) phenotypes. Among 41 slime-producing *E. coli* strains, 7 (17.07%), 14 (34.14%), and 20 (48.78%) strains exhibited high, moderate, and weak levels of biofilm formation, respectively. Fifty-nine (81.94%), and 1(100%) of MDR isolates were assessed as colistin resistant (MIC > 2) and susceptible (MIC ≤ 2) as well. In 26(36.11%) of colistin-resistant isolates and 1(1.38%) of colistin, susceptible isolate *mcr*-1 gene was found. There is no *mcr*- 2 gene was detected in isolates.

**Conclusion:**

The diversity of high antibiotic-resistant rates could be avoided by developing appropriate healthcare policies and community awareness. Alarming resistance rates were observed in colistin and ampicillin, which should be taken into account in therapy guidelines.

## Introduction

Antibiotics have always played an important role in taking care of people’s health all over the globe. Their success in limiting so many infections is undeniable; however, immethodical use of them has led to a rise in antimicrobial-resistant species (AMR). *Escherichia coli* (*E. coli*) in company with other gram-negative bacteria are now one of the main players in this resistance. The rise of AMR leads to a decrease in antimicrobial efficacy, making the treatment procedure more difficult and costly for the patients. What seems to be waiting for us at the end of this path is the appearance of pan-drug resistant species, which will take us back to the pre-antibiotic era [1].

*E. coli* is responsible for most of the gram-negative blood and urinary tract infections (UTI) in humans. Moreover, these gram-negative bacilli are found oftentimes in female genital tracts causing difficulties for pregnant women, such as neonatal sepsis, puerperal and intra-amniotic infections, as well as endocervical and vaginal colonization [1–3].

*E. coli* has a history of antibiotic resistance, as it is proven to be resistant to ampicillin and gentamicin, which were used to cure neonatal infections caused by this specific bacterium. As the resistance towards antimicrobial drugs spreads between bacteria, frequently used drugs such as penicillin’s, beta-lactamase, fluoroquinolones, and aminoglycosides are no longer effective. Hence, we are going back to using older antibiotics like polymyxins. Currently, two of them named polymyxin E (Colistin) and polymyxin B are being used in treatment processes [4]. Colistin may be the last hope to cure infections caused by gram-negative pathogens. The world reporting cases of resistance to this antibiotic is extremely concerning to the experts, and the slow progress of pharmaceutical companies in solving this problem is bringing us closer to a global catastrophe.

Different genes may be the agent of resistance in *E. coli* isolates but according to various studies, *mcr* genes are the most suspected ones [5]. Many studies have been conducted on this matter in animals and poultry specimens, and also a few on human samples. Therefore, this study aims to realize whether the clinically colistin-resistant *E. coli* isolates have mobilized colistin resistance (*mcr-*1, 2) genes in their genome causing them to be resistant to this colistin.

## Materials and methods

### Sample collection and identification of bacterial isolates

In this cross-sectional study, 107 *E. coli* isolates (urine, blood, sputum and stool) were collected (September 2021–March 2022) from clinically hospitalized patient specimens in northwest Iran. Isolates were cultured from transported clinical samples of those patients from different clinical samples suspected to have a clinical infection which hospitalized in the various hospital wards. There is no direct contact with the patients and every laboratory test was conducted on isolated bacteria from transferred clinical samples to the laboratory to further procedure. Blood samples containing citrate anticoagulant were cultured and preserved in tryptic soy broth (TSB) at 37 °C. Initial isolate identification was performed using conventional and standard biochemical microbiology tests and media including gram staining, McConkey agar, sulfide indole motility (SIM), citrate, methyl red (MR), Voges-Proskauer (VP), triple sugar iron (TSI), DNase, urease, gelatinase, lysine decarboxylase, oxidase and catalase (Merck, Germany). The VP and non-motility of the subsequent routine biochemical tests were used as the crucial steps to biochemically determining identity in *E. coli* isolates [6, 7].

### Re-confirmation of *E. coli* isolates by PCR

The PCR test using specific primers also tested and reconfirmed all strains. PCR was accomplished by taking advantage of the corresponding gene to the *E. coli 16S rRNA* according to the previously published [8].

### Antibiotic susceptibility testing

Antibiotic susceptibility testing was performed based on disc diffusion method using imipenem (10 µg), meropenem (10 µg), cefotaxime (30 µg), ceftazidime (10 µg), ciprofloxacin (5 µg), cotrimoxazole (30 µg), gentamicin (10 µg), tetracycline (30 µg), ampicillin + sulbactam (10 µg + 10 µg), piperacillin + tazobactam (100 µg + 10 µg), and levofloxacin (10 µg) (Mast, UK) antibiotics according to clinical laboratory standard institute (CLSI) guideline on Mueller–Hinton Agar plates (Merck, Germany) [9]. Considering the European Centre for Disease Prevention and Control (ECDC) and the Centers for Disease Control and Prevention (CDC) MDR isolates were determined [10].

### ESBL phenotype method

ESBL phenotypes were detected through the results of the Antibiogram according to the referenced article [11]. A phenotypic confirmatory test for ESBL- producing isolate recognition was performed according to CLSI disc diffusion guidelines using cefotaxime (30 µg) and ceftazidime (30 µg) (Mast, UK) in combination with clavulanic acid. After to 24 h of incubation of the Muller-Hinton medium at 37 °C, an over 5 mm diameter increase in the clavulanic acid combined discs compared to the disks without clavulanic acid indicated ESBL production in the cultured bacteria. *K. pneumoniae* ATCC 13883 was used as a control in this step [12].

### In vitro biofilm formation assay

Semi -quantitative biofilm formation assay was done based on the previously published [13]. In short, microtiter plates with *E. coli* bacteria and an equal medium were incubated at 37 °C for 24 h then, the production of biofilm was evaluated taking advantage of the colorimetric assays as follows. After 24-h incubation, the medium was removed and the microtiter plate wells were washed three times with 200 µL of PBS (0.1 M, pH 7.4) buffer, and allowed to dry in the air for 15 min. The microtiter plate wells were stained with 200 µL of 0.4% crystal violet for 10 min at room temperature. The remaining crystal violet stain was removed, and the wells were washed gently three times with 200 µL of PBS buffer. The wells were air-dried for 15 min and the crystal violet in each well was solubilized by adding 200 µL of 33% acetic acid. The plate was read at 630 nm using a microtiter plate reader [13].

### Minimum inhibitory concentration (MIC) of colistin

MIC (minimum inhibitory concentration) for colistin antibiotic was measured using microtiter plate assay in which 0.5-McFarland suspensions of each isolate were cultured in 12 wells and exposed to high to low concentrations of antibiotics according to the kit manufacturer’s protocol, and the minimum concentration of antibiotic that inhibited the growth of microorganisms was got [10]. Serial dilutions of colistin (64–1 µg/ml) were prepared in microtiter plates and a standardized inoculum of 0.5 McFarland was prepared using freshly cultured isolates through the direct colony suspension method. After adding the suspension of the bacteria to the wells, microtiter plates were incubated at 35 ± 2 °C for 20 h. The MIC of colistin as the lowest concentration of colistin that completely inhibits the growth of the organism in the micro-dilution wells was read as detected by the unaided eye [14]. Interpretation of results were conducted based on the previously published ((MIC ≤ 2) sensitive and (MIC > 2) resistant strains) [14].

### Total DNA extraction

Total DNA was extracted based on the previously published study [15]. Briefly, to investigate the presence or absence of genes (*mcr-*1, 2), the total genomic DNA of strains was extracted using the boiling and then DNA extraction kit recommendation (Cinnagen; Cat no. MBK0021).

### Molecular detection of target genes

Standardization of PCR to targeted genes was done according to the published guidelines [15]. The study genes were amplified by PCR using a thermal cycler (Eppendorf, Germany) as follows: PCR reaction was performed in a volume of 25 μl. Master mix Blue (Takara, Japan) and. Finally, the PCR reaction was performed in the thermal cycler following standardization for all target genes. PCR products were then evaluated for the presence of the desired genes (*mcr-1* and *mcr-2*) using electrophoresis on 1.5% agarose gel followed by staining with safe stain [15].

### Statistical analysis

Statistical analysis of the results was accomplished using SPSS 19 (SPSS Inc., IL., Chicago, USA) and a Fisher exact test.

## Results

Out of 107 biochemical-identified*E. coli* isolates recovered from clinical samples, 100 isolates were reconfirmed by targeting *16srRNA* gene. The susceptibility pattern of isolates by disc diffusion method showed that the highest resistance rate was against ampicillin (80.0%) followed by trimethoprim/sulfamethoxazole (60.0%), and the highest susceptibility rates, 98%, 96.8%, and 81.0% were related to imipenem, amikacin, and gentamicin. Detailed data was shown in Table 1.

**Table 1:** Antibiotic susceptibility pattern of isolated strains

	Susceptibility pattern		Resistant
	Sensitive	Intermediate	
Gentamycin	81 (81%)	4 (4%)	15 (15%)
Amikacin	96 (96%)	3 (3%)	1 (1%)
Imipenem	98 (98%)	1 (1%)	1 (1%)
Tetracycline	45 (45%)	0	55 (55%)
Trimethoprim/Sulfamethoxazole	37 (37%)	0	63 (63%)
Ceftazidim	51 (51%)	14 (14%)	35 (35%)
Cefepime	57 (57%)	0	43 (43%)
Ceftriaxone	45 (45%)	0	55 (55%)
Cefotaxime	41 (41%)	1 (1%)	58 (58%)
Ciprofloxacin	40 (40%)		60 (60%)
Ampicilin	18 (18%)	2 (2%)	80 (80%)

Based on the combination disc method, out of 100 *E. coli* isolates, 47 (47%) strains were determined as ESBL phenotypes. Fifty-nine (81.94%), and 1(18.05%) of MDR *E. coli* isolates were assessed as colistin-resistant (MIC > 2) and susceptible (MIC ≤ 2) strains taking advantage of micro dilution test.

Among a total number of 41 slime-producing*. coli* strains, 7 (17.07%), 14 (34.41%), and 20 (48.78%) strains respectively exhibited high ($4O{D}_{C}<OD$), moderate ($O{D}_{C}<OD<2O{D}_{C}$), and weak ($O{D}_{C}<OD<2O{D}_{C}$) levels of biofilm formation, while 59 (59%) were shown to have no biofilm formation capability. Screening of *mcr*-1 gene by using a molecular approach on MDR strains which were detected using the disc diffusion method, we came up with the results. In 26 (36.11%) of *E. coli* isolates with MIC > 2, and 1 (1.38%) isolate with MIC ≤ 2 *mcr*-1 genes were found. There is no *mcr*-2 detected gene in isolated strains. According to the analysis of the results using SPSS, we figured that 37.49% of our MDR- positive species tended to have the *mcr-1* gene in their genome while 81.94% of them (MDR- positives) were resistant to colistin according to our MIC method measures. Around half of these *mcr-1* positive isolates produced biofilm, and the rest belonged to the non-biofilm producing category. There is no *mcr* -2 detected genes in our isolates. Detailed data were reported in Tables 2, 3.

**Table 2:** Frequencies of resistant pattern of utilized antibiotic based on the biofilm formation capability

	Biofilm among *E. coli* resistant to antibiotics			
	Non biofilm	Weak biofilm	Weak biofilm	Strong biofilm
Gentamicin	6 (6%)	2 (2%)	6 (6%)	2 (2%)
Imipenem	0	0	1 (1%)	0
Ciprofloxacin	32 (32%)	12 (12%)	12 (12%)	3 (3%)
Ampicilin	43 (43%)	15 (15%)	15 (15%)	6 (6%)
Ceftazidim	18 (18%)	2 (2%)	8 (8%)	7 (7%)
Cefepime	20 (20%)	4 (4%)	12 (12%)	7 (7%)
Trimethoprim/sulfamethoxanole	33 (33%)	12 (12%)	13 (13%)	6 (6%)
Ceftriaxone	27 (27%)	10 (10%)	12 (12%)	7 (7%)
Tetracycline	30 (30%)	11 (11%)	12 (12%)	7 (7%)
Amikacin	0	0	1 (1%)	0
Cefotaxime	30 (30%)	10 (10%)	12 (12%)	7 (7%)
Colistin	0	0	0	1 (1%)

**Table 3:** Frequency of ESBL phenotypes, *mcr-1* gene and MIC colistin in biofilm forming *E. coli* isolates

Targeted objects				Biofilm formation capability
*mcr-1*	MIC ≤ 2	MIC > 2	ESBL	
3 (27%)	0 (0%)	11 (100%)	9 (45%)	Weak
6 (60%)	0 (0%)	10 (100%)	9 (64%)	Moderate
3 (50%)	1 (16%)	5 (83%)	6 (85%)	Strong
14 (44%)	0 (0%)	33 (100%)	23 (53%)	Non biofilm

## Discussion

*E. coli*, as the main inhabitant of the human gastrointestinal tract (GI), is a causative agent of various infections [16]. The high incidence of Enterobacteriaceae is usual; the isolation rates of mentioned bacteria from suspected urinary tract infections are high. In the current research taking advantage of biochemical recipes, 107 isolates were detected as *E. coli* and following the molecular approach (PCR) using a specific primer, 100 isolates were precisely confirmed as *E. coli*. The relatively high prevalence of *E. coli* in the human clinical infections of the present study may be due to the ubiquitous presence of the bacterium in the hospital environment, its ability to biofilm formation, and finally failure to observe sanitary and disinfection principles [16].

Antibiotic-resistant species tend to be a big threat to humanity as there is a very small chance to survive one when it resists all kinds of treatments. As time passes more species develop resistance to antimicrobial treatments and considering the slow improvements in the pharmaceutical industry, identification, and control of this species may be a tiny silver lining to preventing this issue's disastrous consequences [17]. In our study the rates of resistance were ampicillin (80% Resistant) and imipenem (98% sensitive). In a study conducted by Daoud in 2021., amikacin 98.6% and ampicillin 39.1% were investigated as the highest and lowest resistance rates in isolates [17]. Mostafavi 2019., showed that imipenem (94.9%) and trimethoprim/sulfamethoxazole (31.3%) were as not effective and effective antibiotics on isolates [18]. The reason for these differences can probably be attributed to the type of treatment regimen in hospitals and the way of infection control in the study areas. According to the Disc diffusion results of our antibiogram, ampicillin, and sulfamethoxazole have developed the highest proportion of resistance, respectively.

Based on the localarea, various capabilities of biofilm formation of *E. coli* isolates were observed. In this study, biofilm-producing species formed only 1% of the colistin-resistant *E. coli* samples while the highest percentage of biofilm production appears to belong to ampicillin-resistant species [19]. Yet most antibiotic-resistant species of *E. coli* seem to produce no biofilm.

*E. coli* is one of the most common causative agents of infections around the globe and in our hometown. As a traditional procedure for the prevention and treatment of gastrointestinal gram-negative bacteria such as Enterobacteriaceae infections colistin is widely used in poultry farms and is the last-resort antibiotic for treating multidrug-resistant (MDR) infections [14]. The main part of published studies is focused on colistin-resistant *E. coli* in regional animals. In this survey plasmid-mediated resistance to colistin by targeting the *mcr* -1, 2 in clinically isolated *E. coli* to evaluation of mentioned genes frequencies in human isolates was done. Many studies have been conducted on this matter in animals and poultry, and also a few on human samples. The studies mostly targeted *mcr -1* and not many considered *mcr -2* as a subject of study, which appears to be due to difficulties in its detection [15]. Thus, we decided to study the prevalence of resistance to colistin in *E. coli* infected samples in a certain timeframe and consequently the incidence of *mcr* -1 and *mcr* -2 in resistant species as the most suspected agents of this resistance [14]. After extracting The DNA from our samples, we started setting up our PCR program to achieve the optimum temperature program for both *mcr -1*, 2 to multiply. Although we were able to come up with a plan to run PCR on *mcr -1* successfully there is no detection of *mcr -2* in isolated strains. In conclusion, based on the modifications in the antibiogram interpretation criteria, antibiotic prescription procedure, and wrong traditional prevention and treatment in livestock industries, the susceptibility pattern of *E. coli* is constantly changing. Reconsideration in empirical therapy and restriction in extreme use of antibiotics such as colistin in the livestock industry should be considered.

### Limitations

The genetic relationship between the resistant isolates, investigating any correlations with patient characteristics, and other molecular determinants responsible for colistin resistance of *E. coli* are not aimed and not determined. These are the limitations of the current.

## Data Availability

All results of this study have been classified and maintained by a dissertation in the Zanjan University of medical Sciences. We have indeed provided all raw data on which our study is based. In addition, the datasets analyzed during the current study available from the corresponding author on reasonable request. The data that support the findings of this study are available from an Educational Hospital but restrictions apply to the availability of these data, which were used under license for the current study, and so are not publicly available. Data are however available from the authors upon reasonable request and with permission of Zanjan University of Medical Sciences.
